# Whole exome sequencing highlights rare variants in *CTCF*, *DNMT1*, *DNMT3A*, *EZH2* and *SUV39H1* as associated with FSHD

**DOI:** 10.3389/fgene.2023.1235589

**Published:** 2023-08-22

**Authors:** Claudia Strafella, Valerio Caputo, Sara Bortolani, Eleonora Torchia, Domenica Megalizzi, Giulia Trastulli, Mauro Monforte, Luca Colantoni, Carlo Caltagirone, Enzo Ricci, Giorgio Tasca, Raffaella Cascella, Emiliano Giardina

**Affiliations:** ^1^ Genomic Medicine Laboratory UILDM, IRCCS Santa Lucia Foundation, Rome, Italy; ^2^ Unità Operativa Complessa di Neurologia, Fondazione Policlinico Universitario A. Gemelli IRCCS, Rome, Italy; ^3^ Department of Clinical and Behavioral Neurology, IRCCS Fondazione Santa Lucia, Rome, Italy; ^4^ Istituto di Neurologia, Università Cattolica del Sacro Cuore, Rome, Italy; ^5^ John Walton Muscular Dystrophy Research Centre, Newcastle University and Newcastle Hospitals NHS Foundation Trusts, Newcastle UponTyne, United Kingdom; ^6^ Department of Biomedical Sciences, Catholic University Our Lady of Good Counsel, Tirana, Albania; ^7^ Medical Genetics Laboratory, Department of Biomedicine and Prevention, Tor Vergata University, Rome, Italy

**Keywords:** FSHD, exome, D4Z4, genetics, muscular dystrophy

## Abstract

**Introduction:** Despite the progress made in the study of Facioscapulohumeral Dystrophy (FSHD), the wide heterogeneity of disease complicates its diagnosis and the genotype-phenotype correlation among patients and within families. In this context, the present work employed Whole Exome Sequencing (WES) to investigate known and unknown genetic contributors that may be involved in FSHD and may represent potential disease modifiers, even in presence of a *D4Z4* Reduced Allele (DRA).

**Methods:** A cohort of 126 patients with clinical signs of FSHD were included in the study, which were characterized by *D4Z4* sizing, methylation analysis and WES. Specific protocols were employed for *D4Z4* sizing and methylation analysis, whereas the Illumina^®^ Next-Seq 550 system was utilized for WES. The study included both patients with a DRA compatible with FSHD diagnosis and patients with longer *D4Z4* alleles. In case of patients harboring relevant variants from WES, the molecular analysis was extended to the family members.

**Results:** The WES data analysis highlighted 20 relevant variants, among which 14 were located in known genetic modifiers (*SMCHD1*, *DNMT3B* and *LRIF1*) and 6 in candidate genes (*CTCF*, *DNMT1*, *DNMT3A*, *EZH2* and *SUV39H1*). Most of them were found together with a permissive short (4–7 RU) or borderline/long DRA (8–20 RU), supporting the possibility that different genes can contribute to disease heterogeneity in presence of a FSHD permissive background. The segregation and methylation analysis among family members, together with clinical findings, provided a more comprehensive picture of patients.

**Discussion:** Our results support FSHD pathomechanism being complex with a multigenic contribution by several known (*SMCHD1*, *DNMT3B*, *LRIF1*) and possibly other candidate genes (*CTCF*, *DNMT1*, *DNMT3A*, *EZH2*, *SUV39H1*) to disease penetrance and expressivity. Our results further emphasize the importance of extending the analysis of molecular findings within the proband’s family, with the purpose of providing a broader framework for understanding single cases and allowing finer genotype-phenotype correlations in FSHD-affected families.

## 1 Introduction

The introduction of Next-Generation Sequencing (NGS) into the clinical practice has revolutionized the genetic diagnosis and counseling approach of many Neuromuscular Diseases (NMDs).

In particular, NGS allows detecting a wide range of known genetic alterations associated with NMDs as well as identifying novel genetic variations that can expand the genetic heterogeneity of NMDs (Barp et al., 2021). However, reduced penetrance, variable onset, and expressivity as well as the presence of extra-muscular symptoms in many patients still make the genotype-phenotype correlation of NMDs challenging. Among them, FacioScapuloHumeral Dystrophy (FSHD) represents an excellent example of such level of complexity (Caputo et al., 2022a). FSHD (OMIM #158900, #158901, #619477, #619478) is a skeletal muscle disorder with an estimated prevalence of 1:8000–20.000 (Mostacciuolo et al., 2009; Deenen et al., 2014). A progressive and often asymmetric weakness of facial, shoulder and upper arm muscles are typical features of disease, although abdominal, hip girdle and lower leg muscles are also frequently involved (Preston et al., 2020). Typically, FSHD is inherited as an autosomal dominant disorder, although reduced penetrance and variable expressivity can occur among patients and within families (De Simone et al., 2017; Ricci et al., 2020). FSHD can be distinguished in two forms, namely, FSHD1 and FSHD2, although it can also occur as a compound form of disease (FSHD1+FSHD2). From a genetic perspective, FSHD is associated with the contraction of a macrosatellite repeat array on chromosome 4q35 that is referred to as *D4Z4*. This region normally consists of 11 to >100 Repeated Units (RU) of *D4Z4* elements, whereas it is reduced to 1–10 RU in FSHD1 subjects (Wijmenga et al., 1992). In addition to the *D4Z4* contraction, FSHD has been associated with the presence of pathogenic variants within the *SMCHD1* (18p11.32), *DNMT3B* (20q11.21) and *LRIF1* (1p13.3) genes. These genes have been described as disease modifiers in FSHD1 cases (i.e., with *D4Z4* size of 8–10 RU) or as causative genes in FSHD2 (with a *D4Z4* of 11–20 RU) (Sacconi et al., 2009; Lemmers et al., 2012; van der Boogard et al., 2016; Cascella et al., 2018; Strafella et al., 2019; Hamanaka et al., 2020). In addition, two subtelomeric variants have been identified at chromosome 4, namely, the 4qA and 4qB alleles. Although both are present in the general population, only the 4qA allele is associated with FSHD and it is thereby referred to as “permissive” allele (Lemmers et al., 2010). Moreover, the DNA methylation status of the *D4Z4* locus has been shown to significantly contribute to FSHD severity and penetrance (Lemmers et al., 2015; Himeda et al., 2019). Altogether, these events lead to the relaxation of chromatin conformation, which, in turn, results in the derepression of *DUX4* gene, which is stably transcribed in the presence of the 4qA haplotype containing a polyadenylation signal. In muscle cells, the aberrant expression of DUX4 has been associated with the induction of cell death, oxidative stress and inflammatory pathways, which are thought to be responsible for the progression of muscle damage also *in vivo* (Greco et al., 2020; Cohen et al., 2021).

Despite the progress made in the field, the wide range of mild to severe phenotypes, the occurrence of extra-muscular features, the variable age of onset and progression of disease advocate for FSHD being a complex disorder (Sacconi et al., 2019; Greco et al., 2020). In our practice, approximately 60% of patients with a clinical suspicion of disease are found to be carriers of a reduced *D4Z4* allele compatible with an FSHD1 diagnosis (Zampatti et al., 2019), a percentage that is highly variable and dependent on the experience of the different neurological centers referring the patients. In addition, a reduced *D4Z4* allele in combination with a permissive haplotype has been observed in approximately 3% of the healthy population (Scionti et al., 2012; Ricci et al., 2020). Furthermore, the disease severity has been shown to account for approximately 40% by familial factors and 10% by the *D4Z4* repeat array size (Mul et al., 2018). Given these premises, it is plausible that other (epi)genetic factors contribute to the clinical variability and heterogeneity of FSHD, and the knowledge of these could be important for increasing the accuracy of diagnosis and therefore genetic counseling of patients and families. To this purpose, the present work employed Whole Exome Sequencing (WES) to investigate known and unknown genetic contributors that may be involved in FSHD and may represent potential disease modifiers, even in presence of a *D4Z4* Reduced Allele (DRA). The study included both patients with a *D4Z4* Reduced Allele (DRA) compatible with FSHD diagnosis (≤10RU) and patients with longer *D4Z4* sizes. The study was performed on a large cohort of patients characterized by *D4Z4* sizing, methylation analysis and WES. In case of patients reporting variants of interest from WES analysis, the study was extended to the family members in order to provide a more comprehensive picture of the cases.

## 2 Methods

### 2.1 Study cohort

The study involved 126 Italian patients with clinical signs of FSHD, which accessed to the Genomic Medicine Laboratory-UILDM at the Santa Lucia Foundation IRCCS for the standard molecular diagnosis. The presence of *D4Z4* Reduced Allele (DRA) was evaluated during the diagnostic routine and was utilized to select the study cohort with the purpose of including patients with variable *D4Z4* size. In particular, the molecular assessment of DRA was performed using PFGE and Southern blotting followed by hybridization with specific probes P13-E11 as previously described (Zampatti et al., 2019). The patient’s cohort displayed a variable number of RUs including 15 patients with 1–3 RUs, 80 patients with 4–7 RUs, 7 patients with 8–10 RUs, 2 patients with 11–20 RUs, 6 patients with RUs>20 RUs). Moreover, 16 patients carried two permissive (i.e., both 4qA) DRAs, in the size range between 3 and 20 RU (Supplementary Table S1). The patient’s cohort presented a Female:Male (F:M) ratio of 45:55 and an average age of 52.5 ± 17.7 years. In addition, a cohort of 100 Italian subjects matched for age and sex were included in the study as reference group (Supplementary Table S1).

The clinical evaluation of patients was performed by expert neurologists from Fondazione Policlinico Gemelli IRCCS, using the Clinical Severity Scale (CSS) (Ricci et al., 1999) and the FSHD Clinical Score scale (Lamperti et al., 2010), scores specifically designed and validated to assess disease severity in FSHD patients. Muscle MRI was performed on a 1.5 T scanner (Siemens Magnetom Espree), according to published protocols (Tasca et al., 2014; Tasca et al., 2016; Giacomucci et al., 2020). Upper girdle and lower limb muscle MRI scans were evaluated for the presence of imaging patterns supporting the diagnosis of FSHD (Monforte et al., 2022).

Informed consent was obtained from all the subjects included in the present study.

### 2.2 DNA extraction and methylation analysis

The genomic DNA of patients was extracted from 400 μL of peripheral blood using MagPurix Blood DNA Extraction Kit and MagPurix Automatic Extraction System (Zinexts) according to the manufacturer’s instructions.

Concerning the analysis of methylation, two regions of the *D4Z4* locus were evaluated, namely, the *DR1* (located 1 Kb upstream of the *DUX4* ORF and harboring 29 CpG sites) and the *DUX4*-PAS (containing 10 CpG sites located within the most distal part of the array and including the Polyadenylation Signal, PAS). In particular, the *DUX4*-PAS is specific for the 4q distal region; it is ampliefied only in presence of a 4qA allele and it provides information concerning the presence of a DRA (i.e., FSHD1). The DR1 region is located within each *D4Z4* RU on both chromosomes 4 and 10 and it is highly useful to identify FSHD2 subjects. The DNA from each patient underwent methylation analysis using a protocol based on Bisulfite Sequencing (BSS), Amplified Fragment Length Polymorphism (AFLP) and Machine Learning (ML) described in our previous work (Caputo et al., 2022b). The ML model employs the methylation levels of four CpG sites (*DUX4*-PAS_CpG6, *DUX4*-PAS_CpG3, DR1_CpG1 and DR1_Cpg22) to classify FSHD subjects from non-FSHD ones. Following a specific decision tree (available in Caputo et al., 2022b), the model classify subjects on the basis of specific thresholds of methylation of each CpG site and following a specific order of relevance, that is *DUX4*-PAS_CpG6; *DUX4*-PAS_CpG3; DR1_CpG1; DR1_CpG22.

The characterization of 4q subtelomeric variant was assessed for each converted DNA by means of traditional PCR and electrophoresis. In particular, this PCR employs specific primers for *DUX4*-PAS region, whose amplification is indicative of the presence of at least 4qA allele. The 4qB allele is detected by means of specific primers as well (Caputo et al., 2022b). Three possible 4q configurations were thus distinguished, namely, 4qA/4qA, 4qA/4qB and 4qB/4qB.

### 2.3 Whole Exome sequencing (WES)

Concerning WES analysis, the Illumina^®^ Next-Seq 550 system was utilized. In particular, 30–50 ng/μL of DNA was employed for library preparation by means of Illumina^®^ DNA Prep with Enrichment and Tagmentation kit according to manufacturer’s instructions. The obtained libraries were sequenced at 2 × 100 bp and the sequencing quality of the resulting data was expected to reach a Quality score >30 (Q30) for ∼80% of total called bases. The analysis of variants was performed, focusing on the variants located in genes (*SMCHD1*, *DNMT3B* and *LRIF1*) known to be associated with FSHD as well as genes that may represent candidate novel genes for the disease. The selection of these genes was performed considering their function as epigenetic regulators of *D4Z4*, their location near the *D4Z4* array or genes being targeted by *DUX4*. The list of selected genes was reported in Supplementary Table S2.

The resulting variants were visualized by Integrated Genome Viewer (v.2.7.2) and functionally annotated by means of BaseSpace Variant Interpreter (Illumina, v. 2.15.0.110), using GRCh37 as genome build. Only the variants reporting a minimum coverage of 20X were considered for subsequent analysis. Annotated variants were prioritized considering the type of variants (nonsense, frameshift, missense, splicing); the Minor Allele Frequency (MAF<0.001) in publicly available database (gnomAD) and in the internal reference group; their localization in regulatory regions or protein domains (by consultation of Uniprot and Decipher databases); their pathogenicity scores retrieved by interrogation of bioinformatics prediction tools. In particular, REVEL (Rare Exome Variant Ensemble Learner) is a meta-predictor tool for missense variants that integrates different scores (MutPred, FATHMM v2.3, VEST 3.0, PolyPhen-2, SIFT, PROVEAN, MutationAssessor, MutationTaster, LRT, GERP++, SiPhy, phyloP, phastCons) (Ioannidis et al., 2016). Moreover, Varsite (Laskowski et al., 2020) and Missense3D (Ittisoponpisan et al., 2019) were used to predict the potential effect of missense variants on protein structure and function, whereas variants within the splicing region were analyzed by Human Splicing Finder (v.3.1, https://www.genomnis.com/access-hsf).

The variants with a clinical significance were also confirmed by direct sequencing. To this purpose, the DNA was amplified by PCR, using the AmpliTaq Gold DNA Polymerase (Applied Biosystems) reagents in a total volume of 25 μL, following the manufacturer’s instructions. Successively, direct sequencing of the amplified samples was performed by BigDye Terminator v3.1 Cycle Sequencing Kit (Thermo Fisher Scientific) and run on ABI3130xl (Applied Biosystems). The results were finally analyzed with Sequencing Analysis Software v.6 (Applied Biosystems). In addition, the variants of interest were also subjected to segregation analysis among family members, if available.

Finally, the variants were classified according to the ACMG Standards and Guidelines, which provide a clinical interpretation of variants, discriminating among benign, likely benign, with uncertain significance (VUS), likely pathogenic and pathogenic variants (Richards et al., 2015; Ellard et al., 2020). Bioinformatic online platform (Varsome) and public database collecting data concerning DNA genetic variations (Clinvar, LOVD, Decipher) were also employed as supporting tools for the clinical interpretation of variants. In particular, the PP3 rule was applied following the ClinGen recommendations (Evidence-based calibration of computational tools for missense variant pathogenicity classification and ClinGen recommendations for clinical use of PP3/BP4 criteria) (Pejaver et al., 2022). Concerning the application of PM1 and PP2 rules, Decipher was utilized as supporting tool since it provides helpful and user-friendly tools for assigning such criteria. In particular, Decipher was consulted to visualize the gene and regional constraint (for PP2 application) to missense and loss-of-function variants and the localization of functional domains and regulatory regions (for PM1 rule) of the protein corresponding to the genes of interest. In addition, a “benign cut-off frequency” derived by Varsome was also utilized to compare the frequency of the identified variants in the specific genes and assess the BS1 rule in case of variants with a frequency exceeding the cut-off fixed for each gene.

## 3 Results

The patient’s cohort displayed a variable number of RUs including 15 patients with 1–3 RUs, 80 patients with 4–7 RUs, 7 patients with 8–10 RUs, 2 patients with 11–20 RUs and six patients with RUs>20 RUs). Moreover, 16 patients carried two permissive (i.e., both 4qA) DRAs, in the size range between 3 and 20 RU. All of the patients carried at least a 4qA allele (Supplementary Table S1). The analysis of WES focused the attention on a set of genes selected by their function as epigenetic regulators of *D4Z4*, their location near the *D4Z4* array or genes being targeted by *DUX4* (Supplementary Table S2). Successively, the variants were prioritized according to their frequency, localization into regulatory or protein domains, and bioinformatics prediction.

**FIGURE 1:**
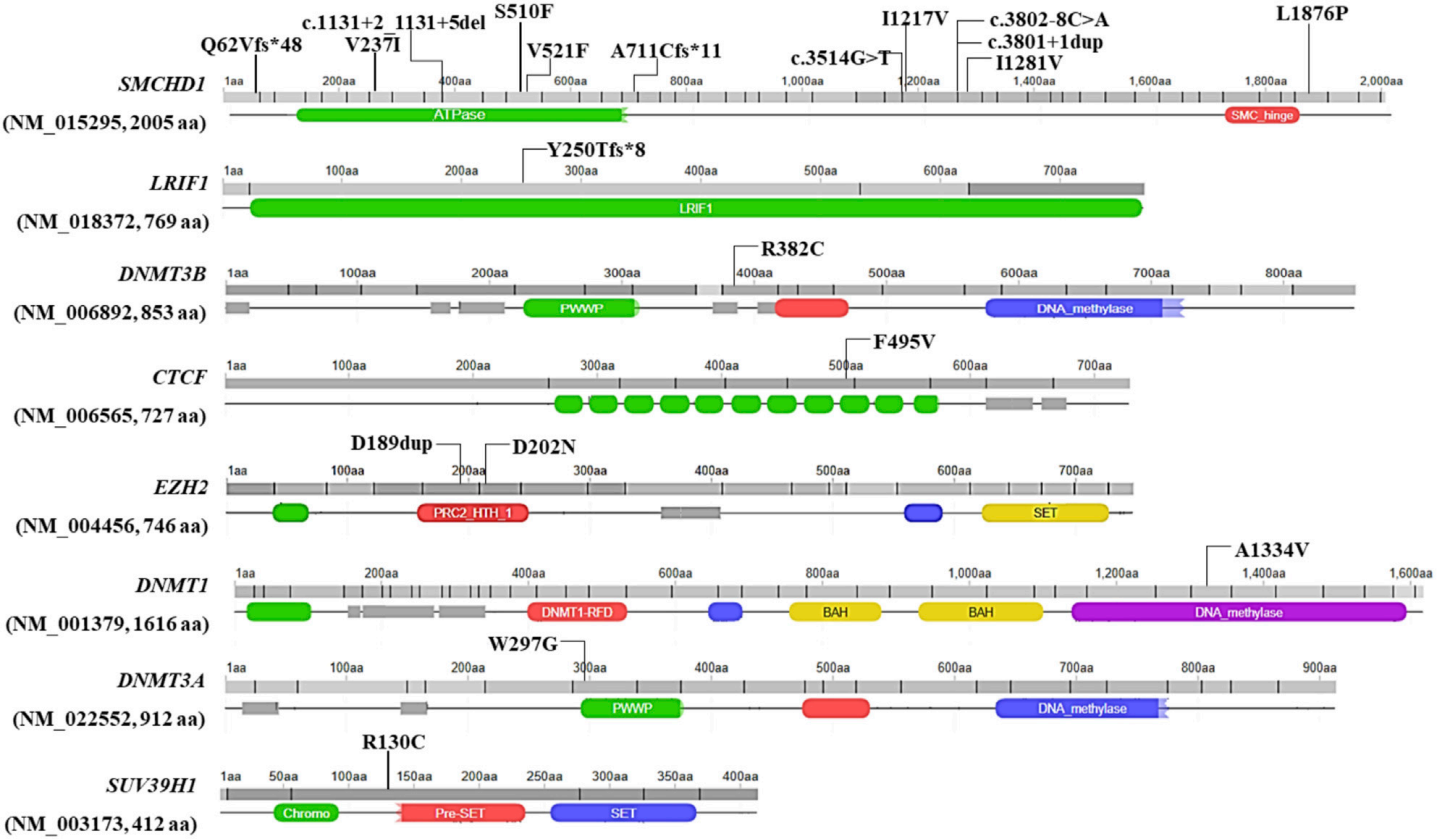
Illustration of the localization of the identified variants at level of the protein sequence. Missense variants are reported with their protein coding nomenclature whereas variants in the splicing regions are indicated with their nucleotide coding. The figure was built by retrieving the domain localization and visualization from Uniprot and Decipher, respectively.

Twenty variants, which were detected in 19 patients with clinical signs of FSHD, emerged from the analysis of WES data. In particular, 14 variants were located in known genes (*SMCHD1*, *DNMT3B* and *LRIF1*), whereas six variants were found in other genes of interest (namely, *CTCF*, *DNMT1*, *DNMT3A*, *EZH2*, *SUV39H1*). None of the variants was observed in the reference group. Interestingly, most of the variants were localized within a specific domain or region of interaction with other factors, thereby suggesting a potential functional effect (Figure 1).

Among the identified variants, 14 were detected in known FSHD2 causing genes (Table 1), namely, *SMCHD1* (*n* = 12), *DNMT3B* (*n* = 1) and *LRIF1* (*n* = 1). These variants were further investigated considering the type (3 frameshift, 4 spicing and 7 missense), frequency in gnomAD and bioinformatics prediction (Table 1). As a result, 12 out of 14 identified variants in known genes were found to be absent or extremely rare in gnomAD database, whereas two variants (*SMCHD1*:c.3841A>G and *DNMT3B*:c.1144C>T) reported a frequency higher than the fixed “benign cut-off frequency.” The use of bioinformatic prediction tools predicted a deleterious or uncertain effect for most variants except for three, which were not reported to have a significant impact on protein function/structure or splicing. Moreover, patients harboring variants in known FSHD genes displayed variable *D4Z4* sizes, including six individuals displaying 4–7 RU, two subjects carrying 9–10 RU, one patient with a *D4Z4*>20RU and four patients with two permissive DRAs (namely, 8 + 6 RU, 5 + 10RU, 8 + 20RU, 13 + 20 RU). Segregation analysis was possible for six cases and showed two patients (FSHD13A and FSHD15A) with *de novo* variants, whereas the remaining ones were inherited or undetermined. Supporting these findings, methylation analysis revealed hypomethylation status consistent with FSHD phenotype in patients harboring such variants, especially in the DR1 region (Table 3). Interestingly, one patient (FSHD1A) harbored a variant in *SMCHD1*, a variant in *LRIF1* and a short DRA (4 RU). Segregation analysis showed that the *SMCHD1* variant was inherited from the unaffected father (FSHD1D), whereas the variant in *LRIF1* was inherited from the affected mother (FSHD1B), together with the short DRA. Methylation analysis in the patient and the family members revealed the lowest methylation levels in the FSHD1A patient with the three events compared to the other family members (Table 3).

**TABLE 1:** Description of the variants identified in known FSHD genes.

Gene name and constraints	Genomic position	Variant type	Variants nomenclature	GnomAD freq	In silico pathogenicity scores	In silico protein prediction	HSF
***SMCHD1* Z (Missense): 3.63 Z (LoF): 8.55 Freq cut-off: 0.00012**	18: 2688462	missense	c.709G>A (p.Val237Ile)	—	moderate pathogenic	The Val residue is very highly conserved	—
						Val237 interacts with ligand (ATP)	
	18: 2739518	missense in splicing region	c.3514G>T (p.Val1172Phe)	—	strong pathogenic	A Val > Phe change is unfavoured in terms of conserved amino acid properties and it has a high “disease propensity” value of 1.20	Broken WT Donor Site. Alteration of the WT Donor site. Most probably affecting splicing
	18: 2743927	splicing	c.3801+1dup	—	na	na	—
	18: 2700830	missense	c.1561G>T (p. Val521Phe)	—	moderate pathogenic	A Val > Phe change is unfavoured in terms of conserved amino acid properties and it has a high ‘disease propensity’ value of 1.20	—
						The Val residue at position 521 is well conserved	
	18: 2700798	missense	c.1529C>T (p.Ser510Phe)	—	moderate pathogenic	A Ser > Phe change is a very large one and might result in a change to the protein’s function	—
						A Ser > Phe change is highly unfavoured in terms of conserved amino acid properties	
						The Ser residue at position 510 is highly conserved	
	18: 2707627	frameshift	c.2129dup (p.A711Cfs*11)	—	na	na	—
	18: 2656258	frameshift	c.182_183dup (p.Q62Vfs*48)	—	na	na	—
	18: 2697122	splicing	c.1131 + 2_1131 + 5	—	na	na	—
			del				
	18: 2747559	missense	c.3841A>G (p.Ile1281Val)	0.000278	supporting benign	not significant impact	—
	18: 2743774	missense	c.3649A>G (p.Ile1217Val)	—	supporting benign	not significant impact	—
	18: 2747512	splicing	c.3802–8C>A	0.000004	na	na	not significant impact
	18: 2784527	missense	c. 5627T>C (p.Leu1876Pro)	—	uncertain	A Leu > Pro change is very highly unfavoured in terms of conserved amino acid properties and it has a very high ‘disease propensity’ value of 3.02	—
***LRIF1* Z (missense): 0.24 Z (LoF): 2.77 Freq: 0.0001**	1: 111494758	frameshift	c.748del (p.Tyr250ThrfsTer8)	—	na	na	—
***DNMT3B*** **Z (Missense):1.5 Z LoF: 5.26** **Freq: 0.00012**	20: 31383232	missense	c.1144C>T (p.Arg382Cys)	0.00053	uncertain	An Arg > Cys change is a very large one and might result in a change to the protein’s function An Arg > Cys change is very highly unfavoured in terms of conserved amino acid properties and it has a high “disease propensity” value of 1.71 The Arg residue at position 382 is poorly conserved	

LoF: Loss of Function. na: not available. HSF: Human Splicing Finder. WT: Wild-Type. Freq: Frequency. Z: z score.

Furthermore, the analysis of WES highlighted the presence of six variants in five genes, namely, *CTCF*, *DNMT3A*, *DNMT1*, *EZH2* and *SUV39H1* (Table 2). The variants were absent or very rare in gnomAD database. All of them were missense, except for one in-frame insertion located in *EZH2*. All of the genes harboring such variations presented a significant constraint (Z score ≥ 3.12) to missense variants, at level of the gene and regions including the identified variants. The application of prediction tools allowed assessing a potential effect of such variants on protein function, structure or splicing (Table 2). Moreover, all of the 6 variants were found in combination with a DRA ranging from 4 to 8 RU and, in two cases (FSHD2A and FSHD5A), with an additional permissive (i.e., 4qA) DRA < 20 RU. Segregation analysis among the available family members showed that the identified variants were found in affected individuals together with a DRA. Moreover, the assessment of methylation levels in families harboring such variants revealed hypomethylated profiles mostly consistent with the clinical status of family members (Table 3).

**TABLE 2:** Description of the variants identified in candidate genes.

Gene name and constraints	Genomic position	Variant type	Variants nomenclature	GnomAD frequency	In silico pathogenicity scores	In silico protein prediction	HSF
***CTCF* Z (Missense): 4.44 Z (LoF): 5.079 Freq: 0.0001**	16: 67660583	missense	c.1483T>G (p.Phe495Val)	not found	moderate pathogenic	A Phe > Val change is unfavoured in terms of conserved amino acid properties and it has a high ‘disease propensity’ value of 1.29. The Phe residue at position 495 is very highly conserved	—
***EZH2* Z (Missense): 4.68 Z (LoF): 5.808 Freq: 0.0001**	7: 148525892	inframe insertion	c.566_568dup (p.Asp189 dup)	0.000004	moderate pathogenic	na	Alteration of auxiliary sequences: significant alteration of ESE/ESS motifs ratio. New Acceptor splice site: activation of a cryptic Acceptor site and potential alteration of splicing
	7: 148525853	missense	c.604G>A (p.Asp202Asn)	0.000004	supporting benign	not-significant impact	—
***DNMT1* Z (Missense): 4.99 Z (LoF): 8.20 Freq: 0.0001**	19: 10249229	missense	c.4001C>T (p.Ala1334Val)	0.00005	uncertain	The Ala residue at position 1334 is very highly conserved. This substitution disrupts all side-chain/side-chain H-bond(s) and/or side-chain/main-chain bond(s) H-bonds formed by a buried ALA residue	—
***DNMT3A* Z (Missense): 3.45 Z (LoF): 1.521 Freq: 0.00062**	2: 25470585	missense	c.889T>G (p. Trp297Gly)	0.000014	moderate pathogenic	A Trp > Gly chain is a very large one and might well result in a change to the protein’s function	—
						A Trp > Gly change is highly unfavoured in terms of conserved amino acid properties and it has a very high ‘disease propensity’ value of 2.80	
						The Trp residue at position 297 is highly conserved. This substitution disrupts all side-chain/side-chain H-bond(s) and/or side-chain/main-chain bond(s) H-bonds formed by a buried Trp residue. This substitution results in a change between buried and exposed state of the target variant residue. TRP is buried and Gly is exposed	
***SUV39H1* Z (Missense): 3.49 Z (LoF): 4.333 Freq: 0.0001**	X: 48558704	missense	c.421C>T (Arg141Cys)	not found	uncertain	A change from an Arg > Cys side chain is a very large one and might well result in a change to the protein’s function. An Arg > Cys change is very highly unfavoured in terms of conserved amino acid properties and it has a high “disease propensity” value of 1.71. The Arg residue at position 130 is fairly well conserved	

LoF, Loss of Function; na, not available; HSF, Human Splicing Finder; WT, Wild-Type; Freq, Frequency; H, Hydrogen; ESE/ESS (Exonic Splicing Enhancer/Exonic Splicing Silencer) Z, z score.

All molecular and clinical data concerning the families harboring variants in known and candidate genes for FSHD have been reported in Tables 3, 4, respectively.

**TABLE 3:** Molecular characterization of FSHD families harboring the variants identified by WES. The column reporting the *D4Z4* size shows the shortest permissive (4qA) allele compatible with the disease for patients 4qA/4qB considering that it is the only one permissive for FSHD. Concerning patients with 4qA/4qA, it has been reported the shortest allele compatible with FSHD, although those ones carrying both *D4Z4* alleles with a size <20 RU have been reported since they both could contribute to disease in these cases. The family link has been reported taking the proband as reference subject. The methylation data refer to the method described in Caputo et al. (2022b) that combines the methylation levels of four CpG sites (*DUX4*-PAS_CpG6, *DUX4*-PAS_CpG3, DR1_CpG1 and DR1_Cpg22) with machine-learning pipeline to classify FSHD subjects from non-FSHD ones (herein referred as to CTRL). Following a specific decision tree (available in Caputo et al., 2022b), the model classify subjects on the basis of specific thresholds of methylation of each CpG site and following a specific order of relevance, that is *DUX4*-PAS_CpG6; *DUX4*-PAS_CpG3; DR1_CpG1; DR1_CpG22. For more details concerning the method employed for methylation analysis, please refer to the article (Caputo et al., 2022b).The utilized thresholds are reported in brackets in each CpG site column. The decision nodes indicate the step of the decision tree utilized by the ML model. RU: Repeated Unit. ML: Machine Learning. CTRL: non-FSHD.

Family ID	ID patient (family link)	Status	*D4Z4* size (RU)	4q	Segregation analysis	*DUX4*-PAS (CpG 6) (≤78%)	*DUX4*-PAS (CpG 3) (≤0.34)	DR1 (CpG 1) (≤0.53)	DR1 (CpG 22) (≤0.99)	Decision Node	ML prediction
FSHD 1	A (proband)	affected	4	A/A	*SMCHD1*:c.5627T>C *LRIF1*:c.748del	57%	27%	48%	80%	3	FSHD
	B (mother)	affected	4	A/A	*LRIF1*:c.748delT	70%	31%	70%	95%	3	FSHD
	C (brother)	unaffected	>20	A/A	*SMCHD1*:c.5627T>C *LRIF1*:c.748del	94%	34%	51%	92%	7	FSHD
	D (father)	unaffected	>20	A/A	*SMCHD1*:c.5627T>C	91%	39%	71%	96%	10	CTRL
FSHD 2	A (proband)	affected	5 + 12	A/A	*CTCF*:c.1483T>G	67%	24%	47%	85%	3	FSHD
	B (son)	unaffected	12	A/B	*CTCF*:c.1483T>G	70%	21%	50%	80%	3	FSHD
	C (son)	unaffected	12	A/B	*CTCF*:c.1483T>G	82%	28%	58%	90%	8	CTRL
	D (son)	affected	5	A/B	Negative	34%	18%	54%	78%	3	FSHD
	E (husband)	unaffected	>20	B/B	Negative	—	—	—	—	10	—
FSHD 3	A (proband)	affected	5	A/A	*EZH2*:c.566_568dup	78%	38%	75%	100%	3	FSHD
	B (daughter)	unaffected	>20	A/A	*EZH2*:c.566_568dup	100%	41%	57%	91%	10	CTRL
	C (daughter)	affected	5	A/A	*EZH2*:c.566_568dup	82%	45%	75%	100%	11	CTRL
	D (husband)	unaffected	>20	A/A	Negative	93%	41%	60%	89%	10	CTRL
FSHD 4	A (proband)	affected	8	A/B	*EZH2*:c.604G>A	48%	15%	64%	64%	3	FSHD
	B (son)	na	8	A/A	Negative	92%	37%	72%	93%	10	CTRL
	C (daughter)	unaffected	14	A/A	*EZH2*:c.604G>A	86%	18%	28%	47%	7	FSHD
FSHD 5	A (proband)	affected	5 + 16	A/A	*DNMT3A*:c.889T>G	59%	12%	62%	100%	3	FSHD
	B (daughter)	unaffected	11 + 16	A/A	Negative	94%	32%	65%	94%	8	CTRL
FSHD 6	A (proband)	affected	6	A/B	*SUV39H1*:c.421C>T	68%	10%	72%	98%	3	FSHD
	B (son)	affected	6	A/B	Negative	78%	21%	67%	96%	3	FSHD
	C (daughter)	unaffected	6	A/B	*SUV39H1*:c.421C>T	39%	17%	42%	85%	3	FSHD
	D (husband)	unaffected	>20	A/B	Negative	95%	63%	69%	97%	10	CTRL
FSHD 7	A (proband)	affected	4	A/B	*DNMT1*:c.4001C>T	56%	16%	45%	85%	3	FSHD
	B (daughter)	unaffected	17 + 17	B/B	Negative	—	—	—	—	10	—
FSHD 8	A (proband)	affected	5	A/B	*DNMT3B*:c.1144C>T	47%	16%	63%	93%	3	FSHD
FSHD 9	A (proband)	affected	7	A/A	*SMCHD1*:c.709G>A	62%	22%	47%	85%	3	FSHD
FSHD 10	A (proband)	affected	5 + 10	A/A	*SMCHD1*:c.3514G>T	32%	8%	31%	22%	3	FSHD
	B (father)	na	5	A/B	negative	85%	28%	57%	93%	8	CTRL
	C (mother)	na	10	A/B	*SMCHD1*:c.3514G>T	48%	9%	35%	64%	3	FSHD
FSHD 11	A (proband)	affected	13 + 20	A/A	*SMCHD1*: c.3801+1dup	49%	29%	41%	84%	3	FSHD
FSHD 12	A (proband)	affected	6	A/A	*SMCHD1*:c.1561G>T	43%	16%	19%	60%	3	FSHD
FSHD 13	A (proband)	affected	8 + 20	A/A	*SMCHD1*:c.1529C>T (*de novo*)	63%	31%	37%	57%	3	FSHD
	B (father)	na	8	A/B	negative	88%	38%	81%	97%	10	CTRL
	C (mother)	unaffected	20	A/B	negative	94%	67%	76%	97%	10	CTRL
FSHD 14	A (proband)	affected	9	A/A	*SMCHD1*:c.2129 dup	68%	48%	47%	86%	3	FSHD
	B (father)	affected		A/A	*SMCHD1*:c.2129 dup	84%	34%	46%	89%	7	FSHD
FSHD 15	A (proband)	affected	10	A/A	*SMCHD1*: c.182_183dup (*de novo*)	51%	10%	24%	58%	3	FSHD
	B (mother)	unaffected	10	A/A	negative	93%	32%	75%	98%	8	CTRL
	C (father)	unaffected	>20	A/A	negative	100%	39%	73%	100%	11	CTRL
FSHD 16	A (proband)	affected	>20	A/A	*SMCHD1*: c.1131 + 2_1131+5del	45%	15%	35%	66%	3	FSHD
FSHD 17	A (proband)	affected	8 + 6	A/A	*SMCHD1*: c.3841A>G	59%	16%	35%	77%	3	FSHD
FSHD 18	A (proband)	affected	7	A/B	*SMCHD1*: c.3649A>G	44%	11%	54%	90%	3	FSHD
FSHD 19	A (proband)	affected	6	A/B	*SMCHD1*: c.3802–8C>A	54%	19%	39%	75%	3	FSHD
	B (son)	affected	6	A/B	negative	59%	14%	73%	96%	3	FSHD

**TABLE 4:** Clinical characterization of FSHD families harboring the variants identified by WES.

Family ID	ID patient	Sex	Age	Status	CSS	FCS	MRI pattern 1	MRI pattern 2	—
FSHD 1	A (proband)	M	39	affected	3.5	9	yes	yes	—
	B (mother)	F	74	affected	3	na	yes	yes	—
	C (brother)	M	43	unaffected	na	na	—	—	MRI na
	D (father)	M	78	unaffected	na	na	—	—	MRI na
FSHD 2	A (proband)	F	59	affected	3	4	yes	yes	—
	B (son)	M	29	unaffected	na	na	—	—	MRI na
	C (son)	M	32	unaffected	na	na	—	—	MRI na
	D (son)	M	36	affected	1	2	—	—	MRI na
	E (husband)	M	62	unaffected	0	0	—	—	MRI na
FSHD 3	A (proband)	F	58	affected	3.5	8	yes	yes	—
	B (daughter)	F	31	unaffected	0	0	no	no	Normal MRI
	C (daughter)	F	23	affected	1	2	no	no	—
	D (husband)	M	59	unaffected	0	0	—	—	MRI na
FSHD 4	A (proband)	M	58	affected	3	6	yes	Yes	
	B (son)	M	28	na	na	na	—	—	MRI na
	C (daughter)	F	27	unaffected	0	0	—	—	MRI na
FSHD 5	A (proband)	M	80	affected	4	4	yes	Yes	—
	B (daughter)	F	57	unaffected	—	—	—	—	MRI na
FSHD 6	A (proband)	F	79	affected	4	7	yes	Yes	—
	B (son)	M	51	affected	1	2	no	No	—
	C (daughter)	F	48	unaffected	0	0	—	—	MRI na
	D (husband)	M	76	unaffected	na	na	—	—	MRI na
FSHD 7	A (proband)	M	64	affected	3.5	7	—	—	UG MRI na
	B (daughter)	F	37	unaffected	na	na	—	—	MRI na
FSHD 8	A (proband)	F	27	affected	1	1	yes	no	—
FSHD 9	A (proband)	F	62	affected	4	8	yes	yes	—
FSHD 10	A (proband)	M	20	affected	1.5	3	yes	yes	—
	B (father)	M	56	na	na	na	—	—	MRI na
	C (mother)	F	49	na	na	na	—	—	MRI na
FSHD 11	A (proband)	F	75	affected	na	na	—	—	MRI na
FSHD 12	A (proband)	M	35	affected	2.5	6	yes	yes	—
FSHD 13	A (proband)	F	47	affected	3.5	na	yes	yes	—
	B (father)	M	71	na	na	na	—	—	MRI na
	C (mother)	F	68	unaffected	na	na	—	—	MRI na
FSHD 14	A (proband)	M	na	affected	3	8	yes	yes	—
	B (father)	M	50	affected	0.5	1	no	no	Normal MRI
FSHD 15	A (proband)	M	34	affected	1	2	yes	yes	—
	B (mother)	F	66	unaffected	na	na	—	—	MRI na
	C (father)	M	66	unaffected	na	na	—	—	MRI na
FSHD 16	A (proband)	F		affected	3	na	yes	yes	—
FSHD 17	A (proband)	M		affected	na	na	—	—	MRI na
FSHD 18	A (proband)	M	56	affected	4	na	—	—	UG MRI na
FSHD 19	A (proband)	F	54	affected	4	7	—	—	UG MRI na
	B (son)	M	23	affected	1.5	na	yes	yes	—

CSS, Clinical Severity Score; FCS, FSHD Clinical Score; na, not available; UG, upper girdle; MRI pattern 1: trapezius involvement and bilateral subscapularis muscle sparing; MRI pattern 2: trapezius involvement, bilateral subscapularis and iliopsoas sparing and asymmetric involvement of upper and lower-limb muscles.

Finally, all the variants identified in known and candidate genes were subjected to ACMG classification, which allowed identifying five Pathogenic variants, 7 Likely pathogenic, 7 VUS and one Likely Benign variant (Supplementary Table S3).

Altogether, these results showed variants in *SMCHD1* as one of the most frequent genetic alterations in this study together with *D4Z4* contraction, whereas variants in *LRIF1* and *DNMT3B* appeared as rarer events, although they may co-occur together with short *D4Z4* contraction and potentially contribute to phenotype variability. Importantly, the WES analysis identified variants in *CTCF*, *DNMT3A*, *DNMT1*, *EZH2* and *SUV39H1*, which have not been described in patients and families with FSHD before and, thus, they may represent novel candidate genetic modifiers for the disease. These variants were found in combination with a DRA, supporting the possibility that different genes can contribute to disease heterogeneity in presence of a FSHD permissive background.

## 4 Discussion

The comprehension of the mechanisms underlying the complex molecular background of FSHD is an area of active research. On this subject, recent studies described transcriptomic and proteomic markers associated with FSHD clinical severity and progression in muscle and blood (Banerji et al., 2019; Corasolla Carregari et al., 2020; Wong et al., 2020; Banerji et al., 2022). In addition, several studies highlighted DNA hypomethylation as a hallmark of disease (Hartwerk et al., 2013; Gaillard et al., 2014; Huichalaf et al., 2014; Calandra et al., 2016; Haynes et al., 2018; Lemmers et al., 2019; Roche et al., 2019; Gould et al., 2021; Banerji et al., 2022; Caputo et al., 2022b; Erdmann et al., 2022; Hiramuki et al., 2022). In this scenario, the identification of *SMCHD1*, *DNMT3B* and *LRIF1* as causative or modifier genes in FSHD1 and FSHD2 laid the foundations for considering FSHD as a complex disease, in which multiple genes are likely to contribute to the disease heterogeneity and variability (Caputo et al., 2022a) To this regard, NGS approaches are the ideal tool to allow the simultaneous investigation of known FSHD causing (*SMCHD1*, *DNMT3B* and *LRIF1*) and other potential candidate gene modifiers. Given these premises, the present study employed WES to investigate known and unknown genetic contributors that may be involved in FSHD, even in presence of a DRA. As a result, the analysis of WES data highlighted 20 variants in 19 patients with clinical signs of FSHD (15% of the total patients’ cohort), including five Pathogenic variants, 7 Likely pathogenic, 7 VUS and one Likely Benign variant (Supplementary Table S3). Among them, 14 variants were detected in known FSHD genes (namely, *SMCHD1*, *LRIF1* and *DNMT3B*). In this case, *SMCHD1* appeared as the most frequently altered gene harbouring 12 variants. As expected, the variants were located throughout the entire gene (Figure 1) and were found to impact protein structure/functioning (in the case of missense variants mostly located in the ATPase domain of *SMCHD1*) or alter splicing and create Premature Termination Codon and truncated proteins (in the case of frameshift, stop-gained or splicing variants) (Table 1). These findings were in line with previous studies (Lemmers et al., 2019; Strafella et al., 2019) highlighting the ATPase domain as one of the most frequently affected domain by FSHD variants, especially by missense ones. Concerning Loss of function (LoF) variants identified in this study (namely, frameshift and intronic variants located in ±1-2 of splice site), they were scattered throughout the gene (Figure 1), consistently with other studies (Lemmers et al., 2019; Sacconi et al., 2019; Strafella et al., 2019; Giacomucci et al., 2020).

Segregation analysis was possible only for six patients carrying *SMCHD1* variants, among which two cases displayed *de novo* variants, whereas the remaining ones were inherited or undetermined (Table 3). Concerning the association with *D4Z4* size, 6 individuals carrying *SMCHD1* variants displayed 4–7 RU (that is clearly in the FSHD1 range); Two subjects showed a 9–10 RU DRA (borderline/short FSHD1 fragments); 1 patients had a *D4Z4*>20 RU (that is in the normal range) and 4 patients revealed two permissive (i.e., 4qA/4qA) DRAs (namely, 5 + 10 RU, 8 + 6 RU, 8 + 20 RU and 13 + 20RU). This result is consistent with the fact that *SMCHD1* can act as causative or modifier gene for FSHD (Lemmers et al., 2012; Sacconi et al., 2019; Strafella et al., 2019). In addition to previous studies, the present work highlighted six cases harbouring both short DRA (4–7 RU) and genetic variants in known FSHD genes (*SMCHD1* and *LRIF1*) and 1 case carrying detrimental variants in *SMCHD1* and *D4Z4* size>20RU, suggesting that these events may contribute to the disease variability among patients and families. In this regard, the FSHD1 family represented a very peculiar case, with the segregation of a short DRA (4 RU), a likely pathogenic variant in *LRIF1* and a VUS in *SMCHD1*. In this family, methylation analysis showed that the patient (FSHD1A) harbouring the three events displayed the lowest methylation levels compared to the affected mother (FSHD1B) and unaffected relatives (Table 3). Moreover, the clinical evaluation revealed a different degree of severity degree between the proband FSHD1A and the affected mother (FSHD1B), supporting a potential combined effect of the *LRIF1* and *SMCHD1* variants in worsening the phenotype (Table 4). Importantly, the *LRIF1* variant was detected at heterozygous state in FSHD1 family, which is in contrast with the other family described in literature (Hamanaka et al., 2020), in which biallelic *LRIF1* variants have been reported together with a permissive (4qA) and a *D4Z4* array of 13 RUs in a patient born from a consanguineous marriage.

The methylation analysis in patients carrying *SMCHD1* variants revealed a marked hypomethylation consistent with FSHD, especially at the level of the DR1 region (Table 3). This finding is consistent with previous studies (Hartwerk et al., 2013; Huichalaf et al., 2014; Caputo et al., 2022b; Hiramuki et al., 2022; Zheng et al., 2023). The striking DR1 hypomethylation supported a functional effect for the identified *SMCHD1* variants, even for those detected in patients carrying a short DRA (in the FSHD1 range). In fact, these patients displayed lower methylation levels compared to their family members carrying the short DRA only. Patient FSHD19A provided a valuable example of such condition, displaying a short DRA (6RU) combined with the *SMCHD1*:c.3802-8C>A variant, for which pathogenicity scores and prediction analysis supported a benign effect. In this case, the methylation analysis in FSHD19A patient revealed a marked reduction of methylation levels in DR1 compared to the other family member (FSHD19B), who displayed the same DRA and 4q subtype but was negative for the *SMCHD1* variant (Table 3). Moreover, other two *SMCHD1* variants were predicted as benign. However, the *SMCHD1*:c.3841A>G displayed a higher frequency than expected (Table 1) and the low methylation levels detected in the patient (FSHD17A) may be due to the presence of two permissive DRAs (8+6 RU). For the *SMCHD1*:c.3649A>G variant (detected in the FSHD18A patient), the methylation analysis did not reveal a marked reduction of DR1 methylation levels. Therefore, the prediction analysis was consistent with additional findings, such as frequency and/or methylation analysis, which equally supported a non-significant effect for both variants in these cases. Overall, the observation of differential methylation profiles in patients harbouring *SMCHD1* variants supports the hypothesis that the methylation analysis is more accurate for assessing the pathogenicity of *SMCHD1* variants compared to bioinformatics prediction tools. In addition, methylation analysis emerges as a useful tool to prioritize subjects in whom the research of variants in FSHD genes should be performed in parallel with *D4Z4* sizing.

Concerning the *DNMT3B* variant, the reported frequency did not support a deleterious effect, and the methylation analysis was lower in the *DUX4*-PAS region, consistently with the presence of a short DRA in the patient (FSHD8A).

**FIGURE 2:**
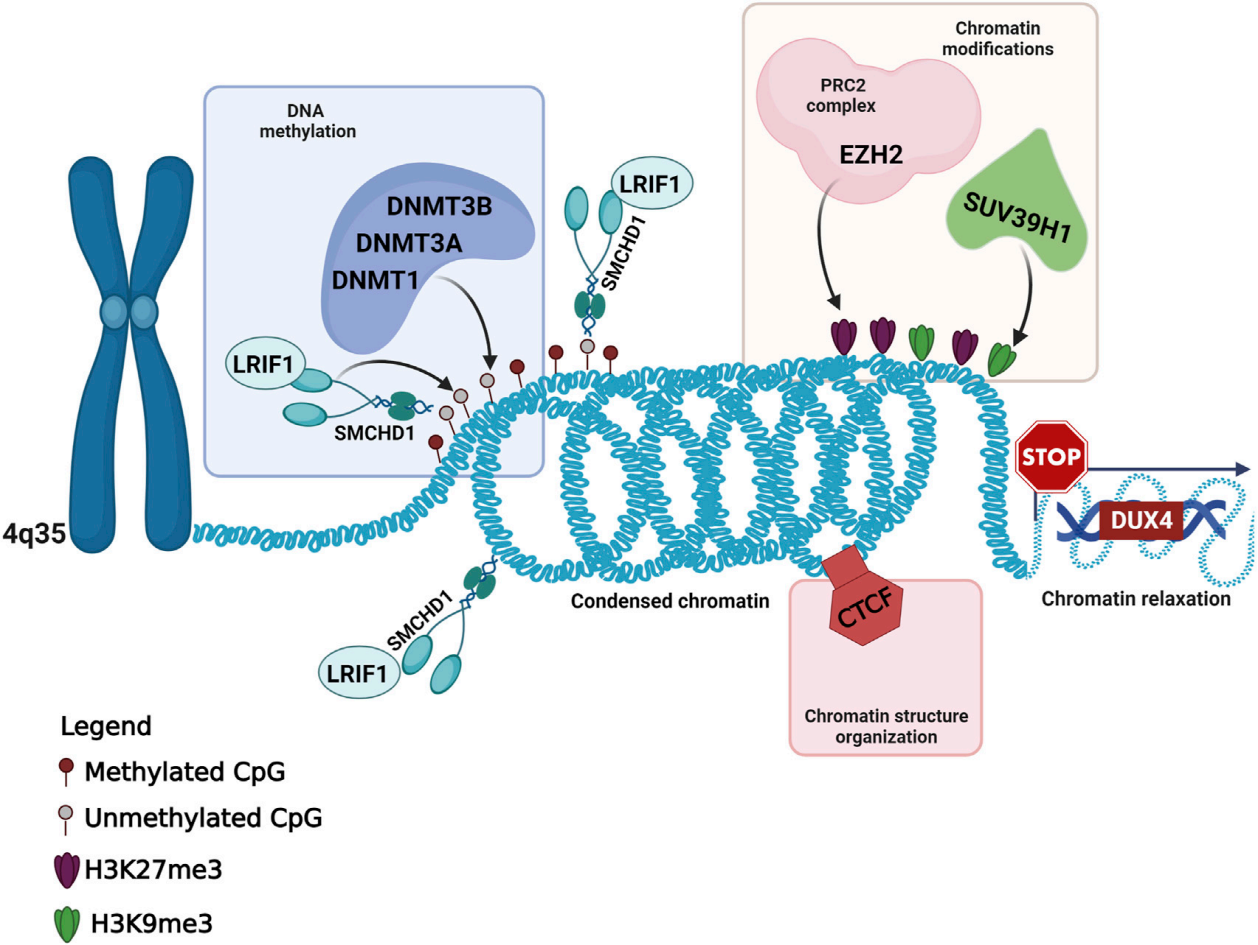
Overview of the known and candidate genes harboring the variants identified in FSHD patients and their role in the maintenance of the *DUX4*-repressive machinery. This figure has been created with Biorender.com.

In addition to the variants detected in known FSHD-causing genes, the present work highlighted the presence of six variants in five genes (*CTCF*, *DNMT1*, *DNMT3A*, *EZH2* and *SUV39H1*), which have been involved in the context of FSHD pathogenesis, although no variant has been described in any of them in FSHD patients. Interestingly, all of these genes have been described as epigenetic regulators of the *D4Z4* locus in the context of FSHD (Zeng et al., 2009; Neguembor et al., 2010; Huichalaf et al., 2014; Himeda et al., 2015). All of them have been found to participate in the maintenance of *DUX4*-repressive machinery, by regulating chromatin modifications (namely, the H3K27me3 and H3K9me3 repressive markers) or DNA methylation (Figure 2). The former are mainly mediated by the activity of EZH2 (which is a member of the Polycomb Repressor Complex 2, PRC2) and SUV39H1, whereas the latter are exerted by DNA Methyltransferases (DNMTs), including DNMT1, DNMT3A and DNMT3B, which are enriched to the FSHD locus and display a redundant role (Huichalaf et al., 2014; Haynes et al., 2018). Moreover, CTCF acts as a multifunctional protein that can mediate transcriptional silencing or activation by creating accessible or inaccessible loops of chromatin at specific sites (Ottaviani et al., 2009; Caputo et al., 2022a). Interestingly, the consultation of Uniprot database revealed that the variants identified in *EZH2* gene were located in the PRC2 complex domain, which interacts with DNMT1, DNMT3A and DNMT3B; the DNMT1 variant was located in the catalytic domain interacting with PRC2; the variant of *DNMT3A* is located in PWWP domain interacting with DNMT1 and DNMT3B. Moreover, the variant identified in *CTCF* has been found to be located in the Zinc Finger 9 (ZF9, C2H2-type 9), which has been involved in the formation and directionality of base-specific interactions between CTCF and its binding sites. Interestingly, the function of ZF9 (together with ZF10 and ZF11) enables CTCF to recognize different DNA sequences across the genome and to promote transcriptional insulation that has been previously described in the pathophysiology of FSHD (Ottaviani et al., 2009; Yin et al., 2017; Xu et al., 2018; Huang et al., 2021). Furthermore, the variant identified in *SUV39H1* was found upstream the pre-SET domain, which plays a structural function in stabilizing the SET domain of the protein. SUV39H1 has been previously proposed as a candidate gene for FSHD, because of its role in mediating the methylation of H3K9, which is critical for HP1γ/cohesion binding (both involved in DUX4 suppression), and for SMCHD1 recruitment, which in turn mediates DNA methylation at *D4Z4* (Zeng et al., 2009; Zeng et al., 2014; Sacconi et al., 2019). The consultation of public databases (Clinvar and LOVD) reported the *EZH2*:c.604G>A and *DNMT1*:c.4001C>T variants as VUS (Clinvar), whereas the other were not described either in Clinvar or in LOVD. In particular, the *EZH2*:c.604G>A was described as VUS for Weaver Smith Syndrome (OMIM #277590), an overgrowth syndrome characterized by accelerated skeletal maturation, characteristic facial appearance and camptodactyly. The *DNMT1*:c.4001C>T was reported as VUS for Hereditary Sensory Neuropathy-Deafness-Dementia Syndrome (OMIM #614116), a degenerative disorder of the central and peripheral nervous systems characterized by sensorineural hearing loss, cerebellar ataxia, narcolepsy and dementia.

Further evidence including functional studies will be needed to finally confirm the modulatory effect of the newly identified variants in FSHD. However, the above-discussed findings and the role of the genes in maintaining the repressive pressure on the *D4Z4* locus suggest that *CTCF*, *DNMT1*, *DNMT3A*, *EZH2* and *SUV39H1* might be further investigated as genes whose alteration contributes to the permissive (epi)genetic environment required to develop FSHD. Interestingly, the patients carrying a variant in one of the candidate genes showed variable *D4Z4* sizes, including permissive DRAs clearly falling in the FSHD1 range (*n* = 3), borderline/short fragments (*n* = 1) and 2 cases with two permissive (i.e., 4qA/4qA) alleles (namely, 5 + 12 RU and 5 + 16 RU) in the FSHD2 range. The integration of these findings with segregation analysis, methylation status, and clinical findings, provided a more comprehensive picture of the probands’ and family phenotype (Table 4).

Methylation analysis in patients and family members harbouring variants in known and candidate genes was mostly consistent with affected/unaffected subjects, although family studies highlighted reduced methylation profiles in five unaffected subjects (FSHD1C, FSHD2B, FSHD4C, FSHD6C and FSHD10C). Of note, all of them were positive to the variants segregating in the families and displayed variable *D4Z4* size, which may affect the penetrance of disease together with unknown mechanisms.

Moreover, we observed higher clinical scores together with lower methylation in *DUX4*-PAS and/or DR1 regions only in FSHD1, FSHD14 and FSHD19 families, whereas the other cases were *de novo* or we did not have enough clinical information or family member to test such a correlation. In general, methylation data appeared to be mostly associated with affected/unaffected status in this study, rather than with FSHD severity as proposed in other studies (Lemmers et al., 2015; Erdmann et al., 2022).

Overall, the variability in methylation profiles and disease severity observed in the families described in this study, could depend on several factors and patients’ characteristics, including 4q configuration (*D4Z4* size, 4q genotype); the variable penetrance of DRA; age; other epigenetic modifications (such as X chromosome inactivation for the FSHD6 family harboring the variant in *SUV39H1*) and still unknown factors that altogether could contribute to the disease severity and clinical variability.

Nevertheless, the assessment of methylation status within the families proved to be a valuable tool not only for discriminating affected subjects, but also for highlighting possible preclinical/asymptomatic conditions among members of the same family who may benefit from a clinical monitoring over time. Although the variants in candidate genes did not show a clear correlation with *D4Z4* size, methylation levels and clinical signs in the investigated patients (FSHD1C, FSHD2C, FSHD3B, FSHD3C, FSHD4C, FSHD6C), this study adds knowledge concerning the possible role of genetic features in the modulation of disease phenotype.

In conclusion, our results further support that FSHD is a complex disease, in which the presence of variations in several known (*SMCHD1*, *DNMT3B*, *LRIF1*) and possibly other genes (*CTCF*, *DNMT1*, *DNMT3A*, *EZH2*, *SUV39H1*) could influence the phenotype, penetrance and severity of disease among patients as well as within the same family. Our results further emphasize the importance of extending the analysis of molecular findings within the proband’s family, with the purpose of providing a broader framework for understanding single cases and allow more accurate genotype-phenotype correlations in FSHD-affected families.

## Data Availability

The datasets presented in this article are not readily available because data obtained from whole exome sequencing are sensitive and. our ethics committee does not authorize the sharing of these data. Requests to access the datasets should be directed to the corresponding author.

## Supplementary material

Supplementary Table S1 List of subjects included in the study. In the First Sheet (“Patient’s cohort”), demographic (sex, anagraphic age), *D4Z4* size and methylation data of patients with clinical signs of FSHD have been reported. The column reporting the *D4Z4* size shows the shortest allele compatible with the disease, although patients carrying an additional permissive (i.e., 4qA) allele <20RU has also been reported. The Second sheet (“reference group”) displays demographic data for the individuals employed as reference population.

Supplementary Table S2 List of genes investigated by analysis of WES data.

Supplementary Table S3 ACMG classification of identified variants in known and candidate genes for FSHD.
