# Supplementary material for: Whole exome sequencing highlights rare variants in CTCF, DNMT1, DNMT3A, EZH2 and SUV39H1 as associated with FSHD
Source: Front Genet. 2023 Aug 22;14:1235589. doi: 10.3389/fgene.2023.1235589 (PMC10477786; doi:10.3389/fgene.2023.1235589)
Supplement: Supplementary file 1 [file Table2.DOCX]

**Table S2:** list of genes investigated by analysis of WES data.

| Genes involved in the epigenetic regulation of *D4Z4* | | |
| --- | --- | --- |
| Gene | **Genomic Location** | **Molecular Function** |
| *SMCHD1* | 18p11.32 | Chromatin remodeler, DNA methylation |
| *DNMT3B* | 20q11.21 | *De novo* DNA methylation |
| *SUV39H1* | Xp11.23 | Histone methyltransferase |
| *SUZ12* | 17q11.2 | Polycomb repressive complex 2 (PRC2) component |
| *EZH2* | 7q36.1 | Polycomb repressive complex 2 (PRC2) component |
| *EED* | 11q14.2 | Polycomb repressive complex 2 (PRC2) component |
| *YY1* | 14q32.2 | *D4Z4* repressive complex component |
| *HMGB2* | 4q34.1 | *D4Z4* repressive complex component |
| *NCL* | 2q37.1 | *D4Z4* repressive complex component |
| *CBX3* | 7p15.2 | *D4Z4* repressor involved in the recognition of methylated histones |
| *LRIF1* | 1p13.3 | *SMCHD1* interactor, transcriptional repressor |
| *SMC1A* | Xp11.22 | Cohesin complex component |
| *RAD21* | 8q24.11 | Cohesin complex component |
| *SMC3* | 10q25.2 | Cohesin complex component |
| *DNMT1* | 19p13.2 | Maintenance of DNA methylation patterns |
| *DNMT3A* | 2p23.3 | *De novo* DNA methylation |
| *DNMT3L* | 21q22.3 | DNA methylation, Interactor for *EZH2*, *DNMT3A* and *DNMT3B*  (promotion/inhibition of DNA methylation) |
| *SETDB1* | 1q21.3 | Histone methyltransferase, transcriptional repressor |
| *CTCF* | 16q22.1 | Chromatin binding factor (either activator or repressor) |
| Genes located near the *D4Z4* array | | |
| Gene | **Genomic Location** | **Molecular Function** |
| *FAT1* | 4q35.2 | Signaling, cell migration and inhibition of proliferation |
| *FRG1* | 4q35.2 | RNA biogenesis |
| *FRG2* | 4q35.2 | Unknown function |
| *DUX4*-target genes | | |
| Gene | **Genomic Location** | **Molecular Function** |
| *PITX1* | 5q31.1 | Transcription factor expressed in limbs and brain |
| *PRAMEF2* | 1p36.21 | Retinoic acid receptor binding |
| *TRIM43* | 2q11.1 | Unknown function |
| *PAX7* | 1p36.13 | Transcription factor involved in myogenesis |
