# Supplementary material for: Whole exome sequencing highlights rare variants in CTCF, DNMT1, DNMT3A, EZH2 and SUV39H1 as associated with FSHD
Source: Front Genet. 2023 Aug 22;14:1235589. doi: 10.3389/fgene.2023.1235589 (PMC10477786; doi:10.3389/fgene.2023.1235589)
Supplement: Supplementary file 2 [file Table3.DOCX]

**Table S3.** ACMG classification of identified variants in known and candidate genes for FSHD.

| Variant | Assessment of criteria | ACMG classification |
| --- | --- | --- |
| *SMCHD1*:  c.709G>A | PM1 (moderate): located in the ATPase activity domain  PM2 (moderate): not found in GnomAD  PP3 (moderate): predicted pathogenesis.  PP2 (Supporting): significant constraint of missense upper Z-score for gene | **Likely pathogenic** |
| *SMCHD1*:  c.3514G>T | PM2 (moderate): absent from GnomAD  PP3 (strong): variant is predicted splicing | **Likely pathogenic** |
| *SMCHD1*:  c.3801+1dup | PVS1 (Very Strong): frameshift mutation  PM2 (Moderate): not found in GnomAD | **Pathogenic** |
| *SMCHD1*:  c.1561G>T | PM1 (moderate): located in the ATPase activity domain  PM2 (moderate): not found in GnomAD  PP3 (moderate): predicted pathogenesis.  PP2 (Supporting): significant constraint of missense upper Z-score for gene | **Likely pathogenic** |
| *SMCHD1*:  c.1529C>T | PM1 (moderate): located in the ATPase activity domain  PM2 (moderate): not found in GnomAD  PP3 (moderate): predicted pathogenesis  PS2 (Strong): de novo | **Pathogenic** |
| *SMCHD1*:  c.2129dup | PVS1 (Very Strong): frameshift mutation  PM2 (Moderate): not found in GnomAD  PP5 (Supporting): reputable source classify as pathogenic | **Pathogenic** |
| *SMCHD1*:  c.182_183dup | PVS1 (Very Strong): frameshift mutation  PM2 (Moderate): not found in GnomAD  PS2 (Strong): de novo variant | **Pathogenic** |
| *SMCHD1*:  c.1131+2_1131+5del | PVS1 (Very Strong): null variant  PM2 (Moderate): not found in GnomAD  PP5 (Supporting): reputable source classify as pathogenic | **Pathogenic** |
| *SMCHD1*:  c. 5627T>C | PM2 (Moderate): absent from GnomAd exomes  PP2 (Supporting): significant constraint of missense upper Z-score for gene | **VUS warm** |
| *SMCHD1*:  c.3649A>G | PM2 (moderate): not found or extremely rare in GnomAD and Internal database  PP2 (Supporting): significant constraint of missense upper Z-score for gene  BP4 (Supporting): position is poorly conserved, computational evidence suggest no impact | VUS cool |
| *SMCHD1*:  c.3802-8C>A | PM2 (moderate): not found or extremely rare in GnomAD  BP4 (Supporting): position is not conserved, computational evidence suggest no impact | VUS cold |
| *SMCHD1*:  c.3841A>G | BS1 (strong): allele frequency is higher than expected  BP4 (supporting): position is poorly conserved, computational evidence suggest no impact | Likely benign |
| *LRIF1*:  c.748del | PVS1 (Strong): frameshift predicted to cause NMD.  PM2 (Supporting): absent or extremely rare in GnomAD. | **Likely pathogenic** |
| *DNMT3B*:  c.1144C>T | BS1 (Supporting): more frequent than expected. | VUS cold |
| *CTCF*:  c.1483T>G | PM1 (Moderate): located in the zinc finger region (C2H2-type 9)  PM2 (Moderate): absent or extremely rare from controls in GnomAD.  PP3 (Moderate): predicted pathogenic.  PP2 (Moderate): missense constraint strongly significant at both gene and regional-level | **Likely Pathogenic** |
| *EZH2*:  c.566_568dup | PM2 (Moderate): absent or extremely rare from controls in GnomAD.  PM1 (Moderate): located in the region interacting with DNMT1, DNMT3A, DNMT3B, and it is located in a compositional bias.  PP3 (Moderate): the position is conserved and HSF predicted a potential alteration of splicing | **Likely pathogenic** |
| *EZH2*:  c.604G>A | PM2 (Moderate): absent or extremely rare from controls in GnomAD.  PM1 (Moderate): located in the region interacting with DNMT1, DNMT3A, DNMT3B, and it is located in a compositional bias.  PP2 (Supporting): missense constraint significant, at both gene and regional-level.  BP4 (Supporting): position is poorly conserved, computational evidence suggest no impact | **VUS warm** |
| *DNMT1:*  c.4001C>T | PM1 (Moderate): located in the catalytic region, interaction with the PRC2/EED-EZH2 complex, C-5 cytosine-specific DNA methylase domain.  PM2 (Supporting) absent or extremely rare from controls in GnomAD.  PP2 (Supporting):significant missense constraint both at gene and regional-level | **VUS warm** |
| *DNMT3A*:  c.889T>G | PM2 (Moderate): absent or extremely rare from controls in GnomAD.  PM1 (Moderate): located in PWWP domain, interaction with DNMT1 and DNMT3B.  PP3 (Moderate): predicted pathogenic.  PP2 (Supporting): significant missense constraint significant at both gene and regional-level. | **Likely Pathogenic** |
| *SUV39H1*:  c.421C>T | PM2 (Moderate): absent or extremely rare from controls in GnomAd.  PP2 (Supporting): significant missense constraint at both gene and regional-level.  PM1 (Supporting): in silico prediction of protein impact support a deleterious effect | **VUS warm** |
